# Loss of tolerance 5 days after discontinuing sulphonamide introduced via desensitization in delayed reaction

**DOI:** 10.31744/einstein_journal/2020RC5002

**Published:** 2019-11-14

**Authors:** Amanda Rocha Firmino Pereira, Marcelo Vivolo Aun, Nathália Coelho Portilho Kelmann, Antônio Abílio Motta, Jorge Kalil, Pedro Giavina-Bianchi

**Affiliations:** 1 Universidade de São Paulo Faculdade de Medicina Hospital das Clínicas São PauloSP Brazil Hospital das Clínicas, Faculdade de Medicina, Universidade de São Paulo, São Paulo, SP, Brazil; 2 Faculdade Israelita de Ciências da Saúde Albert Einstein São PauloSP Brazil Faculdade Israelita de Ciências da Saúde Albert Einstein, São Paulo, SP, Brazil; 3 Universidade de São Paulo Faculdade de Medicina São PauloSP Brazil Faculdade de Medicina, Universidade de São Paulo, São Paulo, SP, Brazil

**Keywords:** Drug hypersensitivity, Trimethoprim, sulfamethoxazole drug combination, Drug eruptions, Desensitization, immunologic, Drug tolerance

## Abstract

The fixed drug eruption is a non-immediate hypersensitivity reaction to drug, characterized by recurrent erythematous or violaceous, rounded, well-defined border plaques, which always appear in the same location every time the culprit drug is administered. The usual practice is to avoid the drug involved and to use a structurally different drug. However, there are situations in which there is no safe and effective therapy. In such situations, desensitization is the only option. We describe the case of a patient who presented fixed eruption due to sulfamethoxazole-trimethoprim, who underwent successful desensitization, but required a repeat procedure twice due to relapse after inadvertent full-dose reintroduction. In non-immediate hypersensitivity reaction to drug, the indication is controversial and there is no technical standardization. Furthermore, the time at which such tolerance is lost after discontinuing the drug involved is unknown. In severe non-immediate reactions of types II and III, desensitization is contraindicated. The patient underwent desensitisation to sulfamethoxazole-trimethoprim three times − the first with recurrence of lesions and the second and third without manifestations, all concluded successfully and with no premedication.

## INTRODUCTION

The fixed drug eruption (FDE) is a drug hypersensitivity reaction (DHR) that is not immediate, characterized by recurrent, rounded, erythematous or violaceous plaques, with well-defined borders, which occur always in the same location, whenever the culprit drug is administered. This reaction may be caused by many different drugs, especially sulfonamides.^(^^1^^–^^7^^)^

The pathogenic mechanisms of FDE are still not well-defined, but TCD8+ cells seem to play an important role at the start of the epidermal lesion, with strong evidence that they permanently reside at the site of the lesions as memory TCD8+ cells.^(^^8^^)^

The habitual practice is to avoid the drug involved and to use a structurally different medication. Nevertheless, there are situations in which there is no safe and effective treatment, and desensitization is the only option. The literature about non-immediate desensitization in DHR is less extensive and more controversial than that for immediate reactions. There are no controlled studies on the theme.^(^^9^^)^

We describe the case of a patient who presented with FDE due to sulfamethoxazole-trimethoprim (SMX-TMP) and underwent successful desensitization, but required two repetitions of the process.

## CASE REPORT

A 68-year-old male patient with Young's syndrome and bronchiectasis as sequelae, with several hospital stays due to exacerbations, presenting with *Nocardia sp* infection in cultures, and for whom the first option of treatment would be SMX-TMP.

Refractory to other antibiotic regimens, and reporting that during hospitalization, in 2002, he presented with erythematous maculae on his body 24 hours after initiating on SMX-TMP. He was referred to the Clinical Immunology and Allergy Division for investigation and diagnostic evaluation. The interval between taking the last tablet of SMX-TMP and the appearance of the lesions was not precise. He was submitted to the oral challenge test in January 2013, with a dose of 400/80mg, which had a negative result for immediate reactions. This was followed by a challenge to assess the likely delayed reaction, which after 7 days of use at home, led to onset of erythematous-violaceous macula on his forearm and lower right limb, which was short-lived and not seen by the medical team.

In the following month, considering the imprecision of the diagnosis, the two-step open oral challenge test was performed. Initially, placebo was administered, which was negative, then SMX-TMP, at a total dose of 1,600/320mg. After 40 minutes, the patient reported pruritus on his hands, more intense on the right hand, and 2 hours later, he presented with erythematous plaque on the medial aspect of the upper right limb, in the same topography as the previous reaction, in 2002. This led to the diagnosis of FDE, and he was treated with oral steroids, with resolution.

**Figure 1:**
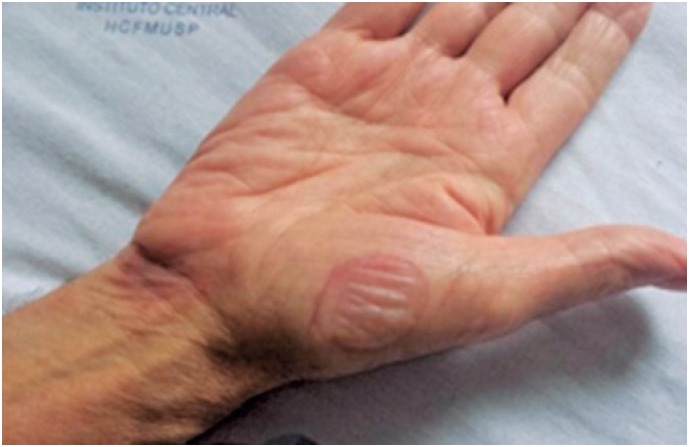
Erythematous bullous lesion on the palmar region

**Figure 2:**
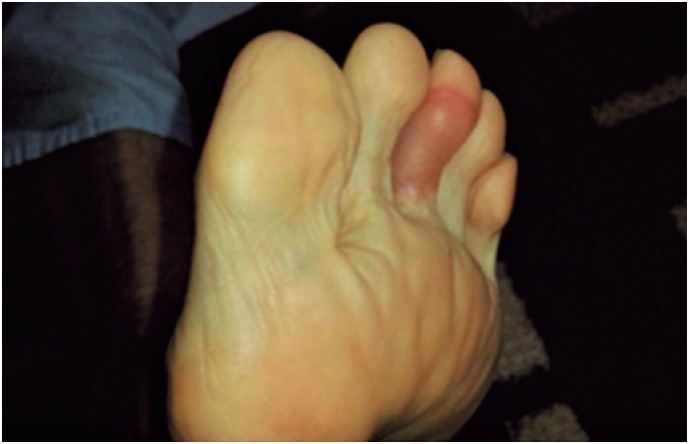
Erythematous violaceous bullous lesion on the plantar region

**Figure 3:**
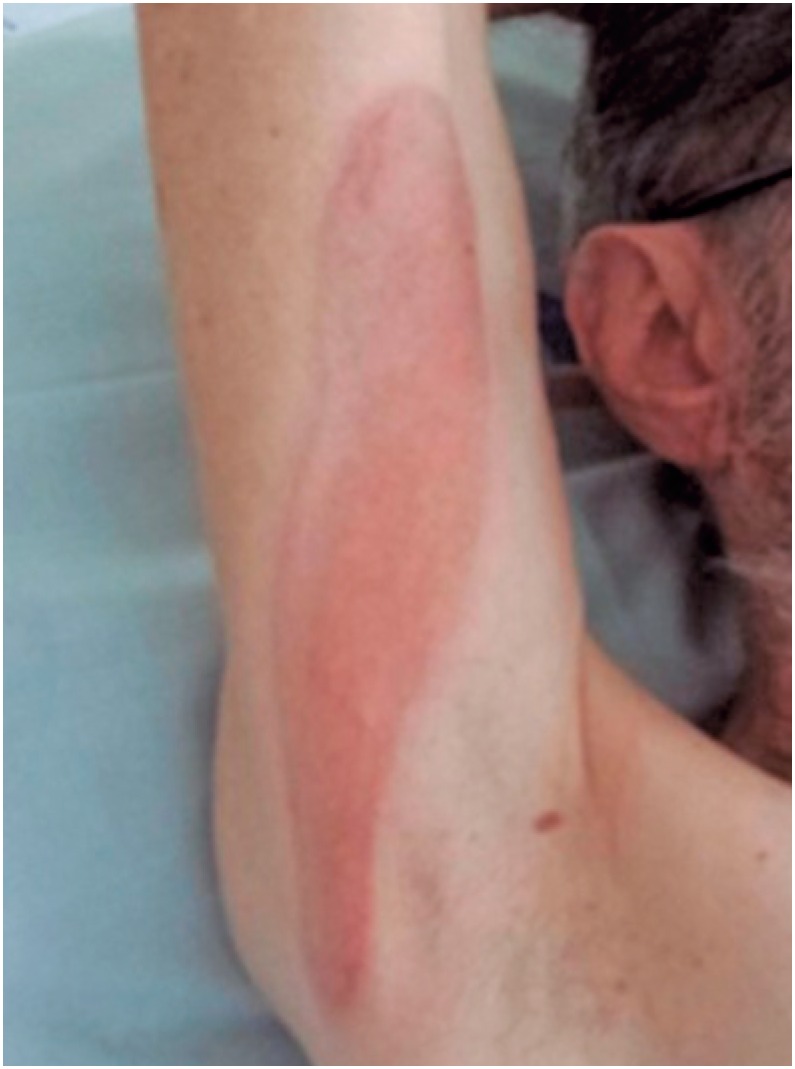
Erythematous bullous lesion on the upper limb

**Figure 4:**
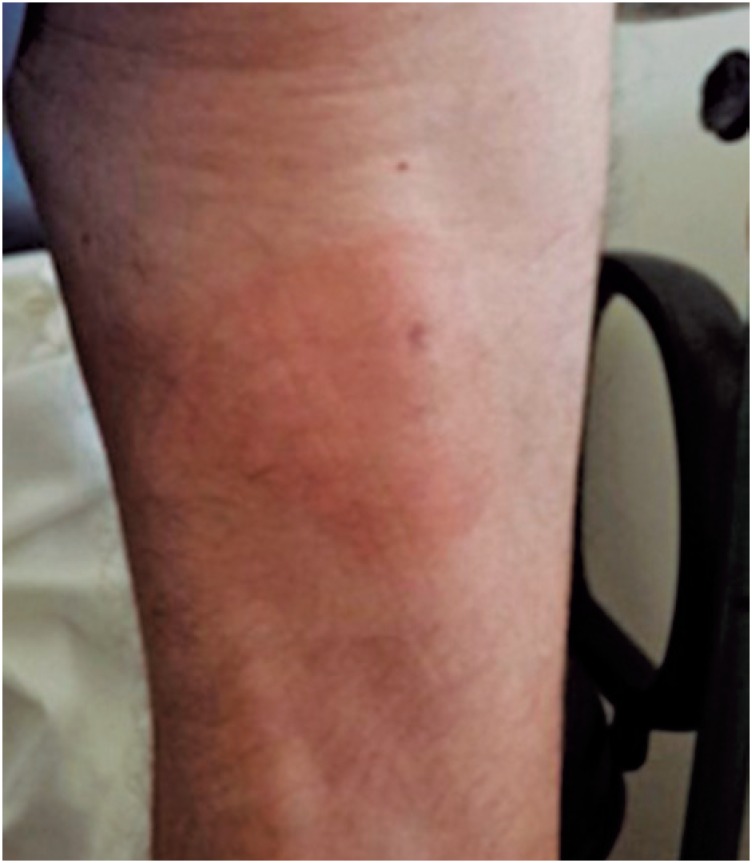
Lesion with residual erythema on the posterior aspect of the lower limb after corticoid treatment

In May 2014, the patient was hospitalized for desensitization with SMX-TMP, and a 10-day protocol was employed (Table 1).^(^^10^^)^ Twenty-four hours after the first administration, the patient presented with a lesion on the medial aspect of the right upper limb, anterior and posterior aspects of the left thigh, and posterior aspect of the right thigh. On the third day, patient evolved with blisters on the palms, right upper limb, and left foot (Figures 1 to 3). The lesions were treated with topical corticoids and desensitization (Figure 4). The procedure was successfully concluded, and the patient maintained the use of 2,400/480 mg a day, with no intercurrent events.

**Table 1:** Protocol for oral desensitization to sulfamethoxazole-trimethoprim using the pediatric suspension (1mL=8mg/40mg) and 80mg/400 mg tablets

Day	Dose	SMX-TMP (mg)
1	1mL of 1:20 of the pediatric suspension of SMX-TMP	0.4/2
2	2mL of 1:20 of the pediatric suspension of SMX-TMP	0.8/4
3	4mL of 1:20 of the pediatric suspension of SMX-TMP	1.6/8
4	8mL of 1:20 of the pediatric suspension of SMX-TMP	3.2/16
5	1mL of the pediatric suspension of SMX-TMP	8/40
6	2mL of the pediatric suspension of SMX-TMP	16/80
7	4mL of the pediatric suspension of SMX-TMP	32/160
8	8mL of the pediatric suspension of SMX-TMP	64/320mg
9	1 tablet of SMX-TMP	80/400mg
10	2 tablets of SMX-TMP	160/800mg

SMX-TMP: sulfamethoxazole-trimethoprim.

In February 2015, the patient maintained the use of SMX-TMP, when he presented with diarrhea, and discontinued himself the medication for 5 days. After the episode improved, he tried to reintroduce the medication, but the lesions recurred. In April 2015, he was admitted for his second desensitization, which, on this occasion, occurred with no intercurrent events and no need of steroids, maintaining a daily use of 2,400/480mg a day.

In June 2015, since he presented with clinical criteria of cure, the patient's treatment for *Nocardia* was discontinued. Thirty days later, he presented new symptoms, and alternative treatments were prescribed, with no clinical control. The patient, on his own initiative, used one tablet of SMX-TMP 400/80mg every 12 hours, and lesions appeared two hours after ingestion of the second tablet. He was referred again on September 2015, and for the third time, desensitization was performed, without complications, and the patient was released with 2,400/480mg a day, with no interruption. All the procedures were performed upon his signing of the Informed Consent Form, using the same protocol.

## DISCUSSION

Desensitization is an induction of transient tolerance. In non-immediate DHR, there is no technical standardization and it is not known how soon this tolerance is lost after discontinuing the drug involved. The time may depend on the type of reaction, drug, and factors related to the patient. Desensitized patients who discontinue the drug need to undergo a new procedure for a fresh induction of tolerance.^(^^9^^)^

In severe non-immediate reactions (Stevens-Johnson syndrome, toxic epidermal necrolysis, induced hypersensitivity syndrome, and acute generalized exanthematous pustulosis), and in type II and type III non-immediate reactions, desensitization is contraindicated.^(^^9^^,^^11^^)^ Occasionally, blisters may develop into the FDE lesions, rarely affecting large body surfaces, and this condition has been called generalized bullous fixed drug eruption (GBFDE).^(^^1^^,^^12^^)^ An Iranian study concluded that many cases of FDE, such as GBFDE, are presented for the first time as FDE with limited lesions, which result in dissemination by repeated exposures to the causative medication. The site most involved in the group treated with antibiotics, especially SMX-TMP, was the upper limb.^(^^6^^)^ GBFDE could be considered a contraindication for desensitization, but it is important to emphasize the absence or scarcity of constitutional symptoms, and no involvement of organs.^(^^1^^,^^12^^)^

The literature has reports of successful desensitization in patients with non-immediate reactions to various drugs.^(^^8^^,^^13^^–^^16^^)^ Nevertheless, these are questionable, since in many cases, the diagnosis of hypersensitivity to the drug was not confirmed by tests, including the challenge test.^(^^9^^)^

A study done in a patient with FDE who was desensitized with allopurinol aimed to evaluate the changes produced on the epidermal damage during the procedure. It is believed that, during desensitization, regulatory cells TCD4+ CD25+ migrate to the lesion site, exerting a suppression effect on the effector function of the T CD8+ cells.^(^^8^^,^^17^^)^

At any reintroduction of the drug in a patient who has experienced a DHR, there is the risk that the same reaction might occur, leading to more extensive manifestations; therefore, it is always important that desensitization occurs in a hospital environment, bearing in mind that complications in non-immediate reactions can occur within hours or even days.^(^^11^^,^^18^^)^

Protocols found for the non-immediate DHR vary in duration from hours to weeks. There seems to be no advantage of the intravenous route over the oral route. There is no consensus on the value of pre-medication and if there is influence on the efficacy of desensitization.^(^^9^^)^ There are reports of patients with HIV who presented with complications during and after desensitization with sulfonamides.^(^^19^^,^^20^^)^

After literature review of the topic, covering the period from 1987 to 2019, this seems to be the first case report of desensitization with sulfa in a patient with GBFDE, which evolved with relapsed reaction after reintroduction of the drug at a therapeutic dose, showing loss of tolerance after discontinuing the drug for a few days. It would be interesting to observe that our patient presented with no manifestations during the second and third desensitization, unlike the first one, perhaps due to early rescue of the memory CD4+ CD25+.

## CONCLUSION

We reported a case in which the patient was submitted to desensitization to sulfamethoxazole-trimethoprim three times, and all were successful without the use of pre-medication. This patient, even while maintaining the use of the medication for months, when it was interrupted, presented with no acquired tolerance. Therefore, in cases of chronic diseases, it would be desirable to sustain the tolerance acquired after desensitization.

## References

[1] 1. Brockow K, Ardern-Jones MR, Mockenhaupt M, Aberer W, Barbaud A, Caubet JC, et al. EAACI position paper on how to classify cutaneous manifestations of drug hypersensitivity. Allergy. 2019;74(1):14-27. 10.1111/all.13562 30028512

[2] 2. Patriarca G, Schiavino D, Buonomo A, Aruanno A, Altomonte G, Nucera E. Desensitization to co-trimoxazole in a patient with fixed drug eruption. J Investig Allergol Clin Immunol. 2008;18(4):309-11. 18714541

[3] 3. Jung JW, Cho SH, Kim KH, Min KU, Kang HR. Clinical features of fixed drug eruption at a tertiary hospital in Korea. Allergy Asthma Immunol Res. 2014;6(5):415-20. 10.4168/aair.2014.6.5.415 PMC4161682 25228998

[4] 4. Emre S, Ahsen H, Aktaş A. Ornidazole-induced fixed drug reaction on sole: case report and review of the literature. Cutan Ocul Toxicol. 2017;36(3):294-6. Review. 10.1080/15569527.2016.1249796 27780370

[5] 5. Can C, Akkelle E, Bay B, Arıcan O, Yalçın O, Yazicioglu M. Generalized fixed drug eruption in a child due to trimethoprim/sulfamethoxazole. Pediatr Allergy Immunol. 2014;25(4):413-5. 10.1111/pai.12204 24750132

[6] 6. Kavoussi H, Rezaei M, Derakhshandeh K, Moradi A, Ebrahimi A, Rashidian H, et al. Clinical Features and Drug Characteristics of Patients with Generalized Fixed Drug Eruption in the West of Iran (2005-2014). Dermatol Res Pract. 2015;2015:236703. 10.1155/2015/236703 PMC4689917 26783389

[7] 7. Kouotou EA, Nansseu JR, Ngono VN, Tatah SA, Zoung-Kanyi Bissek AC, Ndjitoyap Ndam EC. Prevalence and Clinical Profile of Drug Eruptions among Antiretroviral Therapy-Exposed HIV Infected People in Yaoundé, Cameroon. Dermatol Res Pract. 2017;2017:6216193. 10.1155/2017/6216193 PMC5506463 28744306

[8] 8. Teraki Y, Shiohara T. Successful desensitization to fixed drug eruption: the presence of CD25+CD4+ T cells in the epidermis of fixed drug eruption lesions may be involved in the induction of desensitization. Dermatology. 2004;209(1):29-32. 10.1159/000078583 15237264

[9] 9. Scherer K, Brockow K, Aberer W, Gooi JH, Demoly P, Romano A, Schnyder B, Whitaker P, Cernadas JS, Bircher AJ; ENDA, the European Network on Drug Allergy and the EAACI Drug Allergy Interest Group. Desensitization in delayed drug hypersensitivity reactions -- an EAACI position paper of the Drug Allergy Interest Group. Allergy. 2013;68(7):844-52. Review. 10.1111/all.12161 23745779

[10] 10. Absar N, Daneshvar H, Beall G. Desensitization to trimethoprim/sulfamethoxazole in HIV-infected patients. J Allergy Clin Immunol. 1994;93(6):1001-5. 10.1016/s0091-6749(94)70048-6 8006304

[11] 11. Zaouak A, Ben Salem F, Ben Jannet S, Hammami H, Fenniche S. Bullous fixed drug eruption: A potential diagnostic pitfall: a study of 18 cases. Therapie. 2019;74(5):527-30. 10.1016/j.therap.2019.01.009 31006486

[12] 12. Lipowicz S, Sekula P, Ingen-Housz-Oro S, Liss Y, Sassolas B, Dunant A, et al. Prognosis of generalized bullous fixed drug eruption: comparison with Stevens-Johnson syndrome and toxic epidermal necrolysis. Br J Dermatol. 2013;168(4):726-32. 10.1111/bjd.12133 23413807

[13] 13. Audiacana M, Echechipia S, Fernandez E, Urrutia I. Desensitization in a case of fixed drug eruption from allopurinol. Clin Exp Allergy. 1990;(Suppl 1):121.

[14] 14. Garces M, Alonso L, Perez R, Marcos L, Juste S, Blanco J, et al. Successful oral desensitization in a patient with fixed eruption from allopurinol. Allergy. 1995;50 suppl 26:213.

[15] 15. Kelso JM, Keating RM. Successful desensitization for treatment of a fixed drug eruption to allopurinol. J Allergy Clin Immunol. 1996;97(5):1171-2. 10.1016/s0091-6749(96)70275-0 8626998

[16] 16. Umpiérrez A, Cuesta-Herranz J, De Las Heras M, Lluch-Bernal M, Figueredo E, Sastre J. Successful desensitization of a fixed drug eruption caused by allopurinol. J Allergy Clin Immunol. 1998;101(2 Pt 1):286-7. 10.1016/S0091-6749(98)70396-3 9500766

[17] 17. García Rodríguez R, Galindo Bonilla PA, Feo Brito FJ, Gómez Torrijos E, Borja Segade J, Lara de la Rosa P, et al. Chronic desensitization to quinolones in fixed drug eruption. J Investig Allergol Clin Immunol. 2011;21(1):76-7. 21370729

[18] 18. Borish L, Tamir R, Rosenwasser LJ. Intravenous desensitization to beta-lactam antibiotics. J Allergy Clin Immunol. 1987;80(3 Pt 1):314-9. 10.1016/0091-6749(87)90037-6 3040836

[19] 19. Leoung GS, Stanford JF, Giordano MF, Stein A, Torres RA, Giffen CA, Wesley M, Sarracco T, Cooper EC, Dratter V, Smith JJ, Frost KR; American Foundation for AIDS Research (amfAR) Community-Based Clinical Trials Network. Trimethoprim-sulfamethoxazole (TMP-SMZ) dose escalation versus direct rechallenge for Pneumocystis Carinii pneumonia prophylaxis in human immunodeficiency virus-infected patients with previous adverse reaction to TMP-SMZ. J Infect Dis. 200115;184(8):992-7. 10.1086/323353 11574913

[20] 20. Fégueux S, De Truchis P, Balloul H, Maslo C, Matheron S, Coulaud JP. Sulphadiazine desensitization in AIDS patients. AIDS. 1991;5(10):1275-6. 10.1097/00002030-199110000-00028 1786163

