# Supplementary material for: Loss of tolerance 5 days after discontinuing sulphonamide introduced via desensitization in delayed reaction
Source: Einstein (Sao Paulo). 2019 Nov 14;18:eRC5002. doi: 10.31744/einstein_journal/2020RC5002 (PMC6896605; doi:10.31744/einstein_journal/2020RC5002)
Supplement: Translation

# Translation: Perda da tolerância 5 dias após suspensão de sulfonamida introduzida através de dessensibilização por reação tardia

**Authors:** Amanda Rocha Firmino Pereira, Marcelo Vivolo Aun, Nathália Coelho Portilho Kelmann, Antônio Abílio Motta, Jorge Kalil, Pedro Giavina-Bianchi

**Affiliations:** 1 São PauloSP Brasil Hospital das Clínicas, Faculdade de Medicina, Universidade de São Paulo, São Paulo, SP, Brasil; 2 São PauloSP Brasil Faculdade Israelita de Ciências da Saúde Albert Einstein, São Paulo, SP, Brasil; 3 São PauloSP Brasil Faculdade de Medicina, Universidade de São Paulo, São Paulo, SP, Brasil

**Keywords:** Hipersensibilidade a drogas, Combinação trimetoprima e sulfametoxazol, Erupção por droga, Dessensibilização imunológica, Tolerância a medicamentos

## Abstract

A erupção fixa por drogas é uma reação de hipersensibilidade a medicamento não imediata, caracterizada por placas eritematosas ou violáceas, arredondadas, recorrentes, de bordas bem definidas e que aparecem sempre na mesma localização cada vez que o medicamento culpado é administrado. A prática habitual é evitar a droga envolvida e utilizar um medicamento estruturalmente diferente. Contudo, há situações em que não há terapêutica segura e eficaz. Em tais situações, a dessensibilização é a única opção. Descrevemos o caso de um paciente que apresentou erupção fixa por drogas por sulfametoxazol-trimetoprim, tendo sido submetido à dessensibilização com sucesso, mas necessitou repetição do procedimento duas vezes, por recidiva da reação após reintrodução inadvertida em dose plena. Em reação de hipersensibilidade a medicamento não imediata, a indicação é controversa e não há padronização técnica. Além disso, não se conhece o tempo durante o qual essa tolerância é perdida após a suspensão da droga envolvida. Nas reações não imediatas graves e dos tipos II e III, a dessensibilização está contraindicada. O paciente foi submetido a dessensibilização ao sulfametoxazol-trimetoprim por três vezes − a primeira com recorrência de lesões, e a segunda e terceira sem manifestações, sendo todas concluídas com sucesso e sem uso de pré-medicação.

## INTRODUÇÃO

A erupção fixa por drogas (EFD) é uma reação de hipersensibilidade a medicamento (RHM) não imediata, caracterizada por placas eritematosas ou violáceas arredondadas, recorrentes, de bordas bem definidas e que aparecem sempre na mesma localização, cada vez que o medicamento culpado é administrado. Essa reação pode ser causada por muitas drogas diferentes, principalmente pelas sulfonamidas.^(^^1^^–^^7^^)^

Os mecanismos patogênicos da EFD ainda não estão bem definidos, mas as células TCD8+ parecem desempenhar papel importante no início da lesão epidérmica, com fortes evidências de que residam persistentemente no local das lesões como TCD8+ de memória.^(^^8^^)^

A prática habitual é que se evite a droga envolvida e utilize medicamento estruturalmente diferente. Contudo, há situações em que não há terapêutica segura e eficaz, sendo a dessensibilização a única opção. A literatura sobre as dessensibilizações em RHM não imediatas é menos extensa e mais controversa do que para as reações imediatas. Não existem estudos controlados sobre o tema.^(^^9^^)^

Descrevemos o caso de um paciente que apresentou EFD por sulfametoxazol-trimetoprim (SMX-TMP) e foi submetido à dessensibilização com sucesso, mas necessitou repetição do procedimento por duas vezes.

## RELATO DE CASO

Homem de 68 anos, portador de síndrome de Young e bronquiectasias sequelares, com diversas hospitalizações por exacerbação, apresentando, às culturas, infecção por *Nocardia sp*, cuja primeira opção terapêutica seria o SMX-TMP.

Refratário a outros esquemas de antibioticoterapia e com relato de que, durante internação em 2002, apresentara máculas eritematosas no corpo após 24 horas do início do tratamento com SMX-TMP, tendo sido encaminhado à Divisão de Imunologia Clínica e Alergia para investigação e avaliação diagnóstica. Havia imprecisão com relação ao intervalo de tomada do último comprimido de SMX-TMP e o aparecimento das lesões. Foi submetido a teste de provocação oral em janeiro de 2013, com dose de 400/80mg, que resultou negativo para reações imediatas. Foi seguido por provocação para avaliar provável reação tardia, que, após 7 dias do uso domiciliar, cursou com aparecimento de mácula eritêmato-violácea em antebraço e membro inferior direito, fugaz, não visualizada pela equipe médica.

No mês seguinte, diante da imprecisão do diagnóstico, foi realizado novamente o teste de provocação oral aberto em duas etapas. Foi administrado inicialmente o placebo, o qual foi negativo, e SMX-TMP, em dose total de 1.600/320mg. Após 40 minutos, o paciente referiu prurido em mãos, sendo mais intenso em mão direita, e, 2 horas após, apresentava lesão em placa eritematosa na face medial do membro superior direito na mesma topografia da reação de 2002, levando ao diagnóstico de EFD, sendo tratado com corticoide oral com resolução.

Em maio de 2014, foi internado para dessensibilização com SMX-TMP, sendo optado por protocolo de 10 dias (Tabela 1).^(^^10^^)^ Após 24 horas da primeira administração, apresentou lesão na face medial do membro superior direito, face anterior e posterior da coxa esquerda, e face posterior da coxa direita. No terceiro dia, evoluiu com bolhas em palmas das mãos, membro superior direito e pé esquerdo (Figuras 1 a 3). As lesões foram tratadas com corticoide tópico, e prosseguiu-se com a dessensibilização (Figura 4). O procedimento foi finalizado com sucesso, e o paciente manteve o uso de 2.400/480mg ao dia, sem intercorrências.

**Tabela 1:** Protocolo de dessensibilização via oral a sulfametoxazol-trimetoprim utilizando suspensão pediátrica (1mL=8mg/40mg) e comprimido 80mg/400mg

Dia	Dose	SMX-TMP (mg)
1	1mL de 1:20 da suspensão pediátrica de SMX-TMP	0,4/2
2	2mL de 1:20 da suspensão pediátrica de SMX-TMP	0,8/4
3	4mL de 1:20 da suspensão pediátrica de SMX-TMP	1,6/8
4	8mL de 1:20 da suspensão pediátrica de SMX-TMP	3,2/16
5	1mL da suspensão pediátrica de SMX-TMP	8/40
6	2mL da suspensão pediátrica de SMX-TMP	16/80
7	4mL da suspensão pediátrica de SMX-TMP	32/160
8	8mL da suspensão pediátrica de SMX-TMP	64/320mg
9	1 comprimido de SMX-TMP	80/400mg
10	2 comprimidos de SMX-TMP	160/800mg

SMX-TMP: sulfametoxazol-trimetoprim.

**Figura 1:** Lesão eritêmato-bolhosa em região palmar

**Figura 2:** Lesão bolhosa eritêmato-violácea em região plantar

**Figura 3:** Lesão eritêmato-bolhosa em membro superior

**Figura 4:** Lesão com eritema residual em face posterior do membro inferior após tratamento com corticoide

Em fevereiro de 2015, o paciente mantinha uso do SMX-TMP, quando apresentou quadro de diarreia, e suspendeu o medicamento por conta própria por 5 dias. Após melhora do quadro, tentou reintroduzi-lo, porém, houve recorrência das lesões. Em abril de 2015, foi internado para segunda dessensibilização, que, nessa ocasião ocorreu sem intercorrências e sem necessidade de uso de corticoide, mantendo uso diário de 2.400/480mg ao dia.

Em junho de 2015, por apresentar critérios clínicos de cura, teve o tratamento para *Nocardia* suspenso. Após 30 dias, novos sintomas surgiram, sendo instituídos tratamentos alternativos sem controle clínico. Por conta própria, o paciente fez uso do SMX-TMP 400/80mg 1 comprimido a cada 12 horas com aparecimento de lesões duas horas após ingestão do segundo comprimido. Foi reencaminhado em setembro de 2015 e, pela terceira vez, foi realizada a dessensibilização, sem intercorrências, obtendo alta com 2.400/480mg ao dia, sem interrupções. Todos os procedimentos foram realizados mediante assinatura de consentimento livre e informado, utilizando o mesmo protocolo.

## DISCUSSÃO

A dessensibilização é uma indução de tolerância transitória. Em RHM não imediatas, não há padronização técnica e não se conhece em que tempo essa tolerância é perdida após a suspensão da droga envolvida. O tempo pode depender do tipo de reação, droga e de fatores relacionados ao paciente. Pacientes dessensibilizados que suspenderam a droga necessitam ser submetidos a novo procedimento para nova indução da tolerância.^(^^9^^)^

Nas reações não imediatas graves (síndrome de Stevens-Johnson, necrólise epidérmica tóxica, síndrome de hipersensibilidade induzida e pustulose exantemática generalizada aguda) e nas reações não imediatas dos tipos II e III, a dessensibilização está contraindicada.^(^^9^^,^^11^^)^ Ocasionalmente, bolhas podem se desenvolver nas lesões de EFD, raramente abrangendo grandes superfícies corpóreas, entidade que vem sendo então denominada como EFD bolhosa disseminada (EFDBD).^(^^1^^,^^12^^)^ Estudo iraniano concluiu que muitos casos de EFD como de EFDBD são apresentados pela primeira vez como EFD com lesões limitadas, que resultam em disseminação por repetidas exposições ao medicamento causador. O que foi mais observado entre o grupo de antibióticos, especialmente o SMX-TMP, e o local mais envolvido foi o membro superior.^(^^6^^)^ A EFDBD poderia ser considerada contraindicação para a dessensibilização, mas é importante frisar a ausência ou escassez de sintomas constitucionais e o não comprometimento de vísceras.^(^^1^^,^^12^^)^

Na literatura, existem relatos de dessensibilização com sucesso em pacientes com reações não imediatas para diversos medicamentos.^(^^8^^,^^13^^–^^16^^)^ Porém, estes são questionáveis, já que, em muitos, o diagnóstico de hipersensibilidade ao fármaco não foi confirmado por testes, incluindo o de provocação.^(^^9^^)^

Um estudo em um paciente com EFD que foi dessensibilizado com alopurinol buscou avaliar as mudanças produzidas na epiderme lesional durante o procedimento. Acredita-se que, na dessensibilização, as células reguladoras TCD4+CD25+ migram para o local da lesão, exercendo efeito de supressão na função efetora das células T CD8+.^(^^8^^,^^17^^)^

Em qualquer reintrodução de droga em paciente que tenha experimentado uma RHM, há risco de que a mesma reação possa ocorrer e manifestações mais extensas possam ser provocadas, por isto é sempre interessante que a dessensibilização ocorra em ambiente hospitalar, lembrando que as complicações em reações não imediatas podem ocorrer em horas ou mesmo dias.^(^^11^^,^^18^^)^

Os protocolos encontrados para as RHM não imediatas variam em duração de horas a semanas. Parece não haver vantagem da via intravenosa sobre a oral, também não há consenso sobre o valor da pré-medicação e se há influência na eficácia da dessensibilização.^(^^9^^)^ Há relatos de pacientes com HIV que apresentaram intercorrências durante e após a dessensibilização com sulfonamidas.^(^^19^^,^^20^^)^

Após revisão de literatura sobre o assunto, cobrindo o período de 1987 a 2019, este parece ser o primeiro relato de caso de dessensibilização com sulfa em um paciente com EFDBD, que evoluiu com recidiva da reação após reintrodução da droga em dose terapêutica, mostrando a perda da tolerância após suspensão da droga por poucos dias. Seria interessante observar também que nosso paciente não apresentou manifestações durante a segunda e terceira dessensibilização, diferente da primeira, talvez por resgate precoce dos CD4+ CD25+ de memória.

## CONCLUSÃO

Relatamos um caso em que o paciente foi submetido à dessensibilização a sulfametoxazol-trimetoprim por três vezes, sendo todas bem-sucedidas e sem uso de pré-medicação. Este paciente, mesmo mantendo o uso do medicamento por meses, ao interrompê-lo, não apresentou tolerância adquirida. Portanto, nos casos de doenças crônicas, seria desejável sustentar a tolerância alcançada após dessensibilização.
